# A family-oriented antenatal education program to improve birth preparedness and maternal-infant birth outcomes: A cross sectional evaluation study

**DOI:** 10.1186/s12978-019-0776-8

**Published:** 2019-07-16

**Authors:** Yoko Shimpuku, Frida E. Madeni, Shigeko Horiuchi, Kazumi Kubota, Sebalda C. Leshabari

**Affiliations:** 1 Graduate School of Medicine, Kyoto University, 53 Shogoin-kawaharacho, Sakyo-ku, Kyoto, 606-8507 Japan; 2 Magunga District Hospital, P. O. Box 430, Old-Korogwe, Tanga, Tanzania; 3 Graduate School of Nursing Science, St. Luke’s International University, 10-1 Akashi-cho, Chuo-ku, Tokyo, 104-0044 Japan; 4 Department of Biostatistics, Yokohama City University School of Medicine, 3-9 Fukuura, Kanazawa-ku, Yokohama, 236-0004 Japan; 5 School of Nursing, Muhimbili University of Health and Allied Sciences, P. O. Box 65169, Dar es Salaam, Tanzania

**Keywords:** Pregnancy, Childbirth, Birth preparedness, Antenatal education, Male involvement, Africa

## Abstract

**Background:**

In Tanzania, the information on Birth Preparedness and Complication Readiness is insufficiently provided to pregnant women and their families. The aim of this study was to evaluate the maternal and infant outcomes of a family-oriented antenatal group education program that promotes Birth Preparedness and Complication Readiness in rural Tanzania.

**Methods:**

Pregnant women and families were enrolled in a program about nutrition and exercise, danger signs, and birth preparedness. The cross sectional survey was conducted one year later to evaluate if the participants of the program (intervention group) were different from those who did not participate (control group) with respect to birth-preparedness and maternal and infant outcomes.

**Results:**

A total of 194 participants (intervention group, 50; control group, 144) were analyzed. For Birth Preparedness and Complication Readiness, the intervention group participants knew a health facility in case of emergency (OR: 3.11, 95% CI: 1.39–6.97); arranged accompaniment to go to a health facility for birth (OR: 2.56, 95% CI: 1.17–5.60); decided the birthplace with or by the pregnant women (OR: 3.11, 95% CI: 1.44–6.70); and attended antenatal clinic more than four times (OR: 2.39, 95% CI: 1.20–4.78). For birth outcomes, the intervention group had less bleeding or seizure during labour and birth (OR: 0.28, 95%CI: 0.13–0.58); fewer Caesarean sections (OR: 0.16, 95% CI: 0.07–0.36); and less neonatal complications (OR: 0.28, 95% CI: 0.13–0.60).

**Conclusions:**

The four variables were significantly better in the intervention group, i.e., identifying a health facility for emergencies, family accompaniment for facility birth, antenatal visits, and involvement of women in decision-making, which may be key factors for improving birth outcome variables. Having identified these key factors, male involvement and healthy pregnant lives should be emphasized in antenatal education to reduce pregnancy and childbirth complications.

**Trial registration:**

No.2013–273-NA-2013-101.

Registered 12 August 2013.

## Plain English summary

In Tanzania, the information on Birth Preparedness and Complication Readiness (BPCR) is insufficiently provided to pregnant women and their families. Tanzania has been struggling with reducing its maternal mortality ratio: 556/100,000 live births in 2015. As most deaths were from preventable causes, such as postpartum hemorrhage and high blood pressure-related complications, BPCR is essential to timely and safely arrive in health facility. The family-oriented antenatal group education was provided to pregnant women and their families in rural Tanzania. A Tanzanian midwife taught the program on nutrition and exercise, danger signs, and birth preparedness using a picture drama, and the control group received usual care. The same village was visited one year later to evaluate if the program participants were different from those who did not participate with respect to birth-preparedness and maternal and infant outcomes. A total of 194 participants (50 program participants and 144 who did not participate in the program) answered the survey. As a result, the education positively affected BPCR, such as identifying a health facility for emergencies, family accompaniment for facility birth, antenatal care visits, and involvement of women in decision-making. For birth outcomes, the intervention group had less bleeding or seizure during labour and birth, fewer Caesarean sections, and less neonatal complications. Having identified four key factors in BPCR, male involvement and healthy pregnant lives should be emphasized in antenatal education to reduce pregnancy and childbirth complications.

## Introduction

In countries where the maternal mortality ratio remains high, antenatal education to increase Birth Preparedness and Complication Readiness (BPCR) is considered one of the top priorities [1]. BPCR includes birth plans during the antenatal period, such as the birthplace, birth attendant, transportation, health facility for complications, expenses, and birth materials, as well as family coordination to achieve such birth plans. In a meta-analysis of BPCR interventions and birth outcomes [2], exposure to BPCR interventions was associated with a non-significant reduction of 28% in maternal mortality risk (seven studies, RR = 0.72; 95% CI: 0.46, 1.13). Tanzania has been struggling with reducing its maternal mortality ratio despite the continuous efforts; the maternal mortality ratio increased to 556/100,000 live births in 2015 from 454 in 2011 [3, 4]. As most deaths were from postpartum hemorrhage and high blood pressure-related complications [5], identification of pregnancy complications and women’s awareness of the danger signs during the antenatal period are essential [6].

In Tanzania, although increasing, only about half of all pregnant women attend an antenatal clinic more than four times [4]. Moreover, the information provided during antenatal care (ANC) is insufficient. In a study in the Rufiji region, information, education, and communication about the danger signs of pregnancy are reportedly insufficiently provided; only 61% of the clinics provided information despite the national policy recommending the provision of this information in every visit [6].

In the resource-poor settings, antenatal group education is a potential approach because of the limited time for individual counseling at antenatal clinics [7, 8]. Patil et al. conducted an antenatal group counseling program called Centering Pregnancy in Malawi and Tanzania and found the program to be feasible and acceptable among pregnant women. Moreover, it increased respect between healthcare providers and pregnant women [9]. Additionally, Oka et al. suggested the importance of job-aid to provide necessary information to pregnant women during antenatal visits. Job aids were found to be helpful for understanding and recalling information for both health providers and pregnant women [10].

In addition, the influence and decision-making power of the family cannot be ignored in Tanzania. Shimpuku et al. identified perceptional gaps among family members who decide the birthplace and stated that many women considered their husbands as the decision-maker [11]. Tancred et al. reported that women could not arrange transport to go to health facilities owing to financial constraints [12]. Therefore, an antenatal group education that addresses both BPCR and family involvement might be effective for ensuring that women reach health facilities at birth, and consequently serious complications could be identified at the proper time. With little or no research linking group education with birth outcomes, the present research intended to fill that gap.

### Purpose

This study aimed to evaluate an antenatal group education program among pregnant women and their families with respect to birth-preparedness and maternal and infant outcomes in rural villages of Tanzania.

### Hypothesis

The study hypothesis was if Tanzanian pregnant women and their families received a family-oriented antenatal group education, they would (1) have a higher level of BPCR, (2) attend antenatal clinic four or more times, (3) give birth in a health facility, (4) have less complications of women at birth, and (5) have less complications and deaths of infants than those who did not receive the education.

## Methods

### Study design and participants

This research was a cross sectional evaluation study to identify the effects of an antenatal education program on birth preparedness and maternal-infant outcomes (the second phase). All the participants were convenient samples of pregnant women and their families in villages. For the first phase, villagers were recruited to receive an antenatal education program. The details of the process were published elsewhere [13]. For the second phase, the participants in the first phase (the intervention group) were followed after one year. The research team visited the same villages of the first phase and requested the village leaders to announce the present research to both participants of the first phase and those who did not participate in the first phase of the study. The researchers explained the purpose, the content of the second phase, and the ethical considerations. The inclusion criteria were as follows: 16 years old or older, had experienced pregnancy and childbirth in their family including themselves, had no severe physical or psychological illness, and can read Kiswahili.

### Setting

The study was conducted in Korogwe district, which is one of the eight districts in the Tanga region, located in the North Eastern area of the country. Maternal healthcare in Korogwe is provided at one district hospital, three government health centers, one faith-based organization, six private dispensaries, and 41 government dispensaries. Three villages were purposefully selected as the research sites, which were located at least 5 km away from the closest health facility. The distance of villages from health facilities was important as this study focused on preparation of birth and those who live distant from health facilities needed to prepare for birth to be able to seek healthcare when necessary. Thus, if villages were sufficiently close to health facilities, they could access healthcare even if they were not well prepared.

### Family-oriented antenatal group education program

The purpose of this family-oriented antenatal group education program was to promote BPCR and family involvement in pregnancy and childbirth. A picture drama was developed by the research team to convey the story of two pregnant women. The material was first developed in English and then translated by a master-prepared bilingual Tanzanian midwife. The program lasts for approximately 45 min, including explanation of the research, pre-test/post-test, picture drama, and discussion among the participants. The result of the pre-test/post-test was published elsewhere [13]. The Tanzanian midwife led the entire program, reading picture drama and encouraging the participants to talk about the contents in the end.

As the picture drama unfolds, the story shows one woman who had attended an antenatal clinic more than four times. During the antenatal clinic visit, a midwife provided information on appropriate nutrition and exercise, danger signs, and birth preparedness. This pregnant woman and her family had prepared transportation, money, and an accompanying person, and identified a health facility to give birth. When she started having contractions, her family was ready to support her timely trip to a health facility. With the support of a Skilled Birth Attendant (SBA), she gave birth a healthy baby. The other pregnant woman had a family who did not understand the importance of antenatal clinic visits and facility births. She did not visit an antenatal clinic and expected a home birth. When she started having contractions, a Traditional Birth Attendant (TBA) came to support her, but she was having an obstructed labour at that time. They waited for many hours before birth, and then observed that the baby was not breathing after birth. The mother started bleeding after giving birth, thus both the mother and the baby were brought to the hospital, but it was too late to save either one of them. The story illustrated the importance of BPCR and family support, as most household decisions including the birthplace in Tanzania are made by the family members, particularly the husband, and not by the woman [11, 14–16].

### Study outcomes

The primary outcome was whether the BPCR variables of pregnant women and their families were higher in the intervention group than in the control group. As the pre-test/post-test results of the education program was published elsewhere [13], the present report clarifies whether pregnant women had actually prepared for birth according to the BPCR variables, including a visit to an antenatal clinic four times or more. The questions were asked retrospectively in the second phase. The secondary outcomes were as follows: (1) birth in a health facility, (2) women’s complications at birth, and (3) infants’ complications and deaths.

To evaluate the outcomes, the survey items were developed on the basis of the elements of BPCR [1] in English. The survey included demographic information, BPCR, and outcomes of the most recent childbirth in the family including their own childbirth. A Kiswahili-English bilingual translated the English items into Kiswahili. Another Tanzanian researcher who is a PhD holder and is also a Kiswahili-English bilingual conducted the back translation and confirmed the accuracy of the survey items.

### BPCR variables

As for the primary outcome, the BPCR variables included the following elements: desired place of birth; preferred birth attendant; location of the closest facility for birth and in case of complications; funds for any expenses related to birth and in case of complications; supplies and materials necessary to bring to the facility; an identified labour and birth companion; an identified support to look after the home and other children while the woman was away; transport to a facility for birth or in the case of a complications; and identification of compatible blood donors in case of complications. The questions were answerable by yes/no (e.g., “Did you arrange for someone to accompany you or her to go to a health center or a hospital for birth or emergency?”).

### Birth outcome variables

For the secondary outcome, the birth outcome variables included the following: did they give birth in a health facility, did an SBA assisted their birth, were there any complications, was the birth by Caesarean section, was it a live birth, did the baby have any complications, and did they want to give birth again at a health facility. The related questions were to be answered by yes or no (e.g., “Did you or she gave birth at a health center or a hospital?”, “Were there any problems like bleeding or seizure during the labour and birth?”).

### Sample size

The sample size of this study was calculated on the basis of the basic formula with two groups, a two-sided alternative and normal distributions with the same variances. The sample size was calculated as 64 for each group to detect a difference (10 points) between groups at a 5% level of significance with 80% power.

### Data collection

#### The first phase: intervention

During the first phase of the study, the family-oriented antenatal group education program was provided to pregnant women and their families in the intervention group to promote BPCR and family involvement in the villages, as we intended to reach people who were neither attending an antenatal clinic nor planned to give birth at a health facility.

#### The second phase: outcome survey

At the second phase of the study, that is, one year later after providing the education program, we returned to the same three villages and contacted those who attended the education program and those in the control group who did not. Those who agreed to participate answered a survey about their BPCR before childbirth and their behaviors and childbirth outcomes for the most recent childbirth they experienced. For the first phase, the education program was provided in August 2013. For the second phase, the outcome survey was conducted in August 2014.

#### Ethical clearance

Ethical clearance and permissions were obtained from the 1) Institutional Review Board at St. Luke’s International University, Tokyo, Japan (14–040); 2) Director of Korogwe District Council, 3) National Institute for Medical Research (NIMR), Tanzania (NIMR/HQ/R.8/Vol.IX/1604), and 4) Tanzania Commission for Science and Technology (COSTECH) (No.2013–273-NA-2013-101).

### Statistical analysis

For background data, the t-test was used for numerical control of the intervention and control groups (i.e., age and number of children). Pearson’s chi-square test was used for other nominal background data. Those who missed values were excluded from the final analysis, so there was no missing data. For BPCR and outcome variables, the odds ratios (ORs) and 95% confidence intervals (CIs) were also calculated with logistic analysis, comparing the intervention group with the control group. As the number of antenatal visits might affect facility delivery [17, 18], the variable of antenatal visit was used as cofounder in the analysis of outcome variables. Data were analyzed using SPSS ver. 24 (SPSS Inc., Chicago, IL, USA).

## Results

### Sociodemographic characteristics of study population

**Fig. 1:**
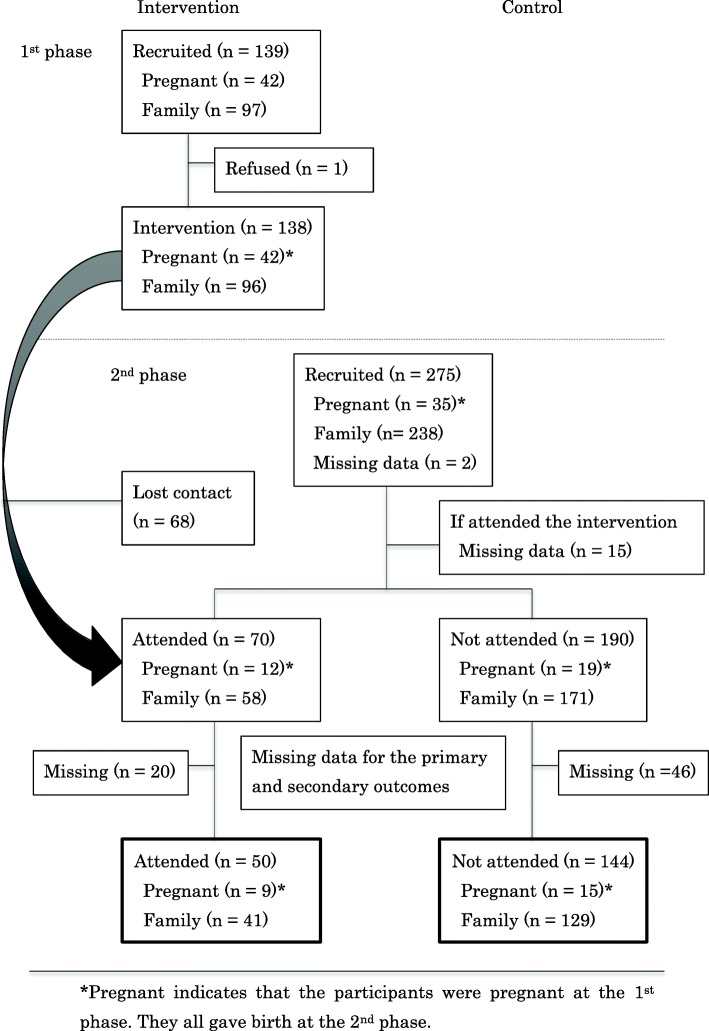
Flow of participants for data collection

Figure 1 shows the flow of recruitment and the number of participants in both the intervention group and control group in each phase. A total of 275 people were recruited and agreed to participate in the second phase. A total of 260 people answered if they participated in the first phase (*n* = 70 in intervention group; *n* = 190 in control group). Furthermore, after eliminating the participants with missing data related to the main outcome variables (*n* = 20 in intervention group; *n* = 46 in control group), the valid responses were 194 (*n* = 50 in intervention group; *n* = 144 in control group). Table 1 shows the sociodemographic variables of both groups. There were no significant differences in the age, gender, marital status, number of children, educational level, occupation, daily expense, and household asset ownership between the two groups based on chi-square test.

**Table 1:** Sociodemographic characteristics

	Intervention group (*n* = 50)		Control group (*n* = 144)		*p-value*
	Mean (SD)	*n* (%)	Mean (SD)	*n* (%)	
Age	39.42 (15.83)		38.42 (15.53)		0.696
Gender					0.672
Women		25 (50)		67 (46.5)	
Men		25 (50)		77 (53.5)	
Marital status					0.487
Married		41 (82)		109 (77.3)	
Not married		9 (18)		32 (22.7)	
Number of children	2.23 (1.65)		2.65 (2.05)		0.241
Educational level					0.109
Below secondary		48 (96)		126 (87.5)	
Secondary and above		2 (4)		18 (12.5)	
Occupation					0.603
Farmer/Engineer		45 (91.8)		125 (87.4)	
Housewife/student		4 (8.2)		18 (12.6)	
Daily expense					0.328
< 5000 Tanzanian Shillings		37 (74)		116 (80.6)	
≥ 5000 Tanzanian Shillings		13 (26)		28 (19.4)	
Household assets ownership					0.860
Low (0–1)		33 (68.8)		95 (67.4)	
High (2+)		15 (31.3)		46 (32.6)	
Birth in family within 12 months					
Women		25		67	0.186
Herself		9 (36)		15 (22.4)	
Others		16 (64)		52 (77.6)	
Men		25		77	0.140
Wife		10 (40)		19 (24.7)	
Others		15 (60)		58 (75.3)	

### Primary outcome: BPCR variables

Table 2 shows the ORs of the BPCR variables in the comparison between the intervention group and the control group. The intervention group participants were more likely to know a health center or a hospital in case of emergency (OR: 3.11, 95% CI: 1.39–6.97, *p* = 0.006), were more likely to arrange someone to accompany them to go to a health center or a hospital for birth or emergency (OR: 2.56, 95% CI: 1.17–5.60, *p* = 0.019), were more likely to decide their birth place (OR: 3.11, 95% CI: 1.44–6.70, *p* = 0.004), and attended an antenatal clinic more than four times (OR: 2.39, 95% CI: 1.20–4.78, *p* = 0.014).

**Table 2:** Odds ratios of BPCR variables in the comparison between the intervention group and the control group (*n* = 194)

Item	OR	95% CI	*p-value*
Knew about the danger signs?	0.64	0.33–1.23	0.182
Planned where she would give birth	0.81	0.37–1.76	0.593
Planned who would attend birth?	0.38	0.11–1.34	0.133
Made arrangement with a nurse, a midwife or a doctor for birth?	0.81	0.39–1.68	0.572
Prepared money for childbirth?	1.60	0.66–3.87	0.295
Prepared transportation to go to a health center or a hospital before labour and birth	0.74	0.37–1.51	0.412
Knew a health center or a hospital in case of emergency	3.11	1.39–6.97	0.006**
Identified a blood donor for childbirth?	0.83	0.43–1.60	0.580
Arranged someone to accompany her to go to a health center or a hospital for birth or emergency	2.56	1.17–5.60	0.019*
Obtained permission from the head of the household to seek skilled care in the event that a birth emergency occurs in his absence	1.60	0.79–3.23	0.190
Arranged a source of household support to provide temporary family care during her absence	1.13	0.50–2.56	0.777
Decided the birthplace with someone or by the pregnant women themselves	3.11	1.44–6.70	0.004**
Attended antenatal clinic more than four times	2.39	1.20–4.78	0.014*

Crude model

* *p* < 0.05, ** *p* < 0.01

### Secondary outcomes: birth outcome variables

Table 3 shows the ORs of the birth outcome variables in the comparison between the intervention group and the control group when adjusted for sociodemographic variables. The intervention group participants were less likely to have problems such as bleeding or seizures during labour and birth (OR: 0.28, 95% CI: 0.13–0.58, *p* = 0.001) and less likely to have a Caesarean section (OR: 0.16, 95% CI: 0.07–0.36, *p* = 0.000). The babies in the intervention group were less likely to have complications (OR: 0.28, 95% CI: 0.13–0.60, *p* = 0.001). Although it was an important birth outcome variable, there was no significant difference in health facility birth (OR: 1.98, 95% CI: 0.95–4.15, *p* = 0.064).

**Table 3:** Odds ratios of the birth outcome variables in the comparison between the intervention group and the control group (*n* = 194)

Item	OR	95% CI	*p-value*
Gave birth at a health center or a hospital	1.96	0.96–3.98	0.064
Gave birth with an SBA	1.68	0.87–3.25	0.123
Had problems such as bleeding or seizure during labour and birth	0.28	0.13–0.58	0.001**
Had Caesarean section	0.16	0.07–0.36	0.000**
Baby was alive when born	2.32	0.99–5.44	0.052
Baby had complication	0.28	0.13–0.60	0.001**

Crude model

* *p* < 0.05, ** *p* < 0.01

As the ‘ANC visit more than 4 times’ is an important factor that affects other outcomes [15, 16], the birth outcome variables were adjusted for ANC visits. Table 4 shows the ORs of the birth outcome variables in the comparison between the intervention group and the control group. The items with statistical significance in Table 3 remained statistically significant after adjustment.

**Table 4:** Odds ratios of the birth outcome variables in the comparison between the intervention group and the control group adjusted for ANC visit (*n* = 194)

Item	OR	95% CI	*p-value*
Gave birth at a health center or a hospital	1.65	0.79–3.48	0.185
Gave birth with an SBA	1.69	0.86–3.32	0.129
Had problems such as bleeding or seizure during labour and birth	0.27	0.13–0.57	0.001**
Had Caesarean section	0.14	0.06–0.31	0.000**
Baby was alive when born	2.21	0.91–5.34	0.079
Baby had complication	0.24	0.11–0.55	0.001**

Adjusted for visit to antenatal clinic more than 4 times

* *p* < 0.05, ** *p* < 0.01

## Discussion

The current study showed that the family-oriented antenatal group education program had a potential significant effect on BPCR, namely, identifying a health facility for emergency, family accompaniment for facility birth, ANC visits, and involvement of women in decision-making as well as maternal/neonatal complications. Firstly, the change in women’s involvement in decision-making for their birthplace had a unique cultural aspect that warrants discussion. In Tanzanian culture, many women lack the decision-making power within the family which hinders their birth preparedness particularly on transportation and birthplace as those require financial preparation [11, 12, 19, 20]. Moshi and Nyamhanga reported that men perceived their role in providing financial support; however, they tended to be less concerned about birthplace because they considered birth as women’s role [21]. Although the Tanzanian Ministry of Health supported the involvement of men in childbirth [22], it has been difficult to involve them in reality owing to traditional roles and lack of knowledge [23–25]. Similarly, though the education of the present study involved husbands and taught them the need of preparation for transportation, there were no statistically significant differences in the actual preparation for transportation despite the fact that women’s involvement in decision-making improved. It suggests emphasizing resource mobilization to men in the education as Moshi and Nyamhanga argued that as long as male partners did not perceive that childbirth contained risks, they did not mobilize financial resources [21]. August et al. suggested potential contributions of community health workers to male involvement from their study in Rufiji and Mkuranga [26]. Additionally, the importance of financial resource mobilization could be supported as Shimamoto and Gipson showed that increase in women’s higher household decision-making power and employment were related to SBA use in Tanzania [27].

Another important aspect of the study was that the intervention group was more likely to attend ANC clinic more than four times. In Tanzania, the rate of ANC attendance for more than four times was still 51% [4]. The number of ANC clinic is important as other study revealed the relationship with neonatal birth outcomes, such as the number of low-birth-weight (LBW) babies, and LBW was associated with stillbirths, low Apgar score, and early neonatal deaths [28]. McMahon et al. reported that birth before arrival of health facility was associated with the low number of ANC visits in rural districts of Morogoro Region [20]. Challaghan-Koru et al. described from their qualitative study in Morogoro that one of the barriers against attending ANC clinic was miscommunication between providers and pregnant women about when and how often they should visit ANC clinic [29]. The success in increasing the number of ANC visits in this study might have been because the education was clear about the number of ANC visits and explained why it was important to visit, i.e., receiving examinations and education from health providers. Oka et al. used the same content and showed the positive effect in communication between healthcare providers and pregnant women [10]. Although facility delivery was not significantly different between two groups in this study, Choe et al. had a congruent result and stated that cultural and family barrier is one of the reason of disconnection between the number of ANC visits and facility delivery in rural Tanzania [30]. As WHO recently changed the number of ANC contacts in the guideline to eight, and they emphasize the importance of women’s positive pregnancy experience, promotion of clear communication between healthcare providers and pregnant women on the number and timing of ANC contacts needs to be continued [31].

Regarding improvement of family members’ accompaniment to health facility, the education had been provided for pregnant women to timely and safely reach a health facility, but it might have influenced quality of care they received at the health facility. Although there was no statistical difference in facility delivery, significant differences were found in self-reported birth complications and Caesarean section between two groups. A Cochrane review showed the continuous support during labor reduced Caesarean section [32]. The intervention group might have had family companion as they are more prepared for family members to accompany pregnant women to health facility. On contrary, Dynes et al.’s study in Kigoma Region reported that clients had significantly greater odds of having a birth companion if they self-reported labor complications (aOR 2.82, 95% CI 1.02–7.81) [33]. However, they discussed that it might be because women having perceptions of risks might request family attendance.

It was also considered that the education could have promoted healthier pregnant lives among the women in the intervention group as the education included nutrition and the appropriate amount of exercise. In fact, the pre-/post-evaluation immediately after the education in the first phase showed significant improvement in understanding nutrition during pregnancy [13]. Promoting healthier pregnancies that reduce the risk of complications is important where human and medical resources are limited, and where transportation is difficult to obtain. For example, maternal deaths in Bangladesh were reduced significantly from 574/100,000 live births in 1990 to 170 in 2013; however, their increase in facility birth rate was limited to 42.1% [34]. According to Alfreen et al., it was estimated that 52% of maternal deaths would have occurred in 2010 in view of the 2001 rates, but these were averted because of decreases in both fertility and risk factors for maternal death [35]. To decrease the high-prevalence risks in Tanzania, such as postpartum hemorrhage and complications related to high blood pressure [5], improving nutrition could be a feasible option. For example, WHO recommends calcium supplementation to avoid hypertensive disorders during pregnancy [36, 37]. A recent study in Tanzania showed that moderate-to-severe anemia (Hb < 90 g/L) was strongly associated with blood loss at birth and the immediate postpartum period, after adjusting for maternal covariates and variables of biological relevance to blood loss [38].

There were several limitations in this study that must be addressed. The convenient sampling might have caused selection bias such that the intervention group participants were more aware of danger signs or healthy pregnant life. As the study was cross sectional, we cannot eliminate potential baseline differences between the groups. The data were all retrieved retrospectively from women and their families, which might have caused recall bias. In addition, as the participants in both the intervention and control groups were from the same villages, cross-contamination of information such as the importance of facility use was possible. In terms of threats to external validity, we collected data in three villages of Korogwe district; therefore, generalizability might be limited when applying the findings to other places.

However, it is equally important to emphasize the key finding and the benefits of the study, that is, the family-oriented antenatal group education program is cost-effective compared with individual counseling or home visits. In the era of SDGs, global health projects are exposed to the question of sustainability [39]. As the present intervention has a potential aspect of sustainability and impact on birth outcomes, the next step is to train local healthcare providers or community health workers to continuously provide this family-oriented antenatal group education for women and their families during pregnancy in villages. This will pave the way for larger studies on BPCR and maternal-infant birth outcomes to be conducted.

## Conclusions

This study revealed the potential positive effects of our family-oriented antenatal group education on four BPCR variables: identifying a health facility for emergencies, family accompaniment for facility birth, ANC visits, and involvement of women in decision-making. The outcomes related to maternal and neonatal complications and Caesarean section were fewer in the intervention group. With the identification of the above key factors, male involvement and promotion of healthier pregnant lives should be emphasized in antenatal education in rural Tanzania to reduce birth complications during pregnancy and childbirth.

## Data Availability

The datasets used and analyzed during the current study are available from the corresponding author upon reasonable request.

## Abbreviations

ANC: Antenatal Care
BPCR: Birth Preparedness and Complication Readiness
LBW: Low-Birth-Weight
SBAs: Skilled Birth Attendants
SDGs: Sustainable Development Goals
TBAs: Traditional Birth Attendants
